# Strand-specific libraries for high throughput RNA sequencing (RNA-Seq) prepared without poly(A) selection

**DOI:** 10.1186/1758-907X-3-9

**Published:** 2012-12-28

**Authors:** Zhao Zhang, William E Theurkauf, Zhiping Weng, Phillip D Zamore

**Affiliations:** 1 Biochemistry and Molecular Pharmacology, and Howard Hughes Medical Institute, University of Massachusetts Medical School, 364 Plantation Street, Worcester, MA, 01605, USA; 2 Program in Molecular Medicine, University of Massachusetts Medical School, 373 Plantation Street, Worcester, MA, 01605, USA; 3 Program in Bioinformatics and Integrative Biology, University of Massachusetts Medical School, 364 Plantation Street, Worcester, MA, 01605, USA

**Keywords:** RNA-Seq, Transcriptome, High throughput sequencing

## Abstract

**Background:**

High throughput DNA sequencing technology has enabled quantification of all the RNAs in a cell or tissue, a method widely known as RNA sequencing (RNA-Seq). However, non-coding RNAs such as rRNA are highly abundant and can consume >70% of sequencing reads. A common approach is to extract only polyadenylated mRNA; however, such approaches are blind to RNAs with short or no poly(A) tails, leading to an incomplete view of the transcriptome. Another challenge of preparing RNA-Seq libraries is to preserve the strand information of the RNAs.

**Design:**

Here, we describe a procedure for preparing RNA-Seq libraries from 1 to 4 μg total RNA without poly(A) selection. Our method combines the deoxyuridine triphosphate (dUTP)/uracil-DNA glycosylase (UDG) strategy to achieve strand specificity with AMPure XP magnetic beads to perform size selection. Together, these steps eliminate gel purification, allowing a library to be made in less than two days. We barcode each library during the final PCR amplification step, allowing several samples to be sequenced in a single lane without sacrificing read length. Libraries prepared using this protocol are compatible with Illumina GAII, GAIIx and HiSeq 2000 platforms.

**Discussion:**

The RNA-Seq protocol described here yields strand-specific transcriptome libraries without poly(A) selection, which provide approximately 90% mappable sequences. Typically, more than 85% of mapped reads correspond to protein-coding genes and only 6% derive from non-coding RNAs. The protocol has been used to measure RNA transcript identity and abundance in tissues from flies, mice, rats, chickens, and frogs, demonstrating its general applicability.

## Background

Strand-specific RNA sequencing (RNA-Seq) provides a powerful tool for transcriptome analysis. Besides measuring transcript abundance across the entire transcriptome, RNA-Seq facilitates *de novo* transcript annotation and assembly, quantification of splice site usage, and identification of mutations or polymorphisms between samples [1-3]. Ribosomal RNAs compose an overwhelming fraction of the total RNA population (>70%) and can occupy most of the sequencing space, leaving little room for investigating other transcripts [4]. The most widely used strategy employs poly (A) selection to enrich RNA polymerase II transcripts, but this strategy cannot be used to study RNAs lacking poly(A) tails or precursor transcripts processed into fragments that have lost their poly(A) tails; for example, 7SL RNA, 7SK RNA, the 5^′^ fragment of Argonaute cleavage products, processed products of PIWI-interacting RNA (piRNA) precursors, and long non-coding RNAs such as Kcnq1ot1 in mammals [5]. Another strategy removes rRNA by hybridization while retaining other non-adenylated RNAs for sequencing [5].

Although RNA can be sequenced directly, without conversion to cDNA, current high throughput technologies for direct RNA sequencing have short read lengths (25 to 55 nt; median 33 nt) and high error rates (4%) [6,7]. Therefore, current strategies for transcriptome analysis all typically convert RNA to cDNA before sequencing [8-11], notwithstanding the artifacts that may result from template switching or structural RNA self-priming [8-10]. The deoxyuridine triphosphate (dUTP) method, one of the leading cDNA-based strategies, provides excellent library complexity, strand specificity, coverage evenness, agreement with known annotation and accuracy for expression profiling [12]. In this method, the RNA is first reverse transcribed into cDNA:RNA using random primers. To synthesize the second cDNA strand, dUTP instead of deoxythymidine triphosphate (dTTP) is used, marking the second cDNA strand for subsequent degradation with uracil-DNA glycosylase (UDG) to preserve strand information [13-15].

**Figure 1:**
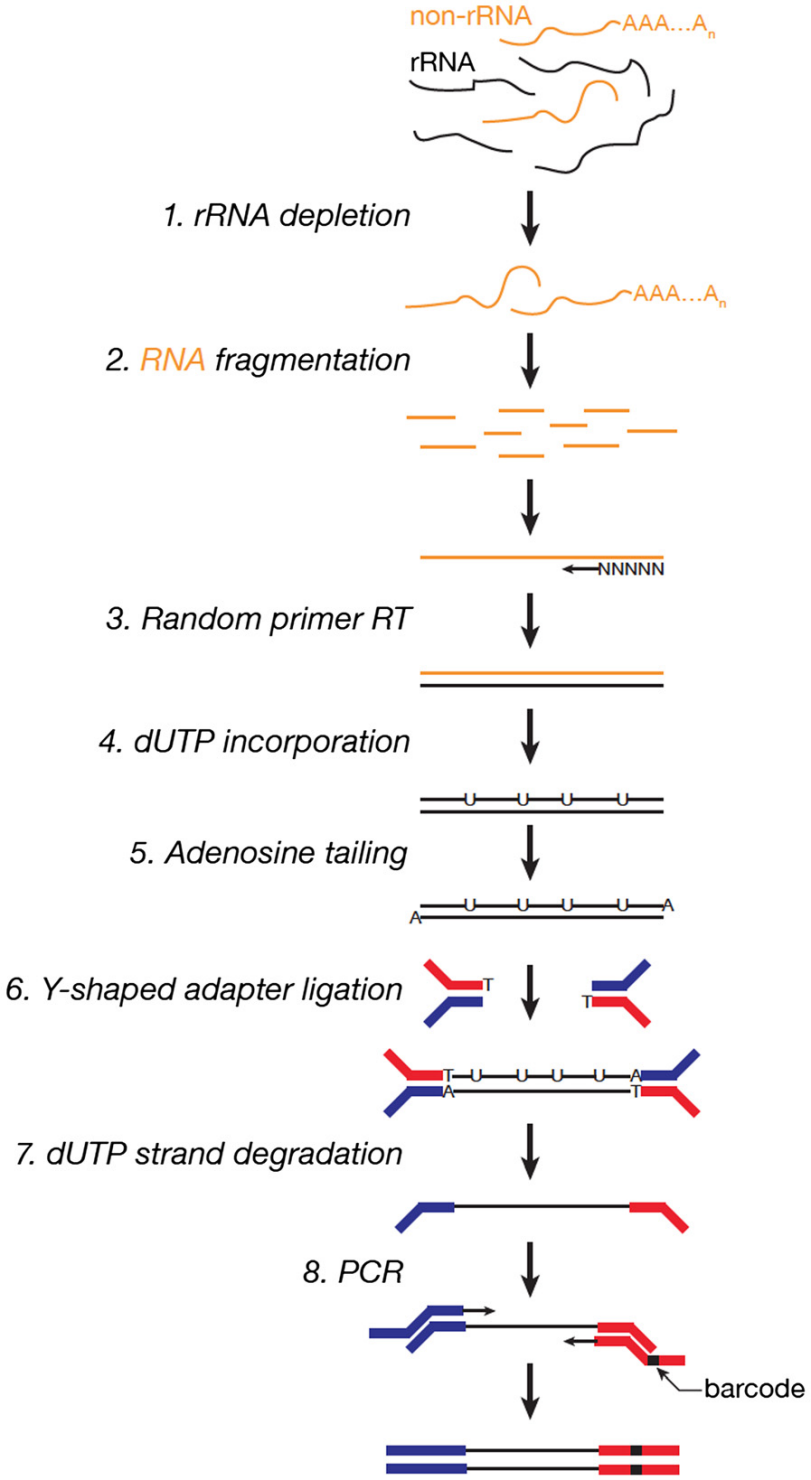
Protocol workflow.

**Figure 2:**
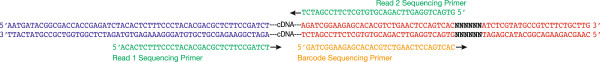
Library and sequencing primer sequences.

Here, we describe a protocol for preparing strand-specific RNA-Seq libraries that combines rRNA removal using the Ribo-Zero rRNA Removal Kit (Epicentre, Madison, WI, USA) and the dUTP method for ensuring strand specificity (Figure 1). Our protocol shows advantages in time saved, cost and performance (Table 1). We replace laborious, time-consuming gel purification steps with AMPure XP beads (Beckman Coulter, Brea CA, USA), whose size-selectivity and efficiency of DNA recovery allow the use of small amounts of starting RNA [16,17]. The high sequencing depth of the Illumina HiSeq 2000 platform (Illumina, San Diego, CA, USA) can easily generate >170 million reads per lane, allowing multiple barcoded samples to be pooled and sequenced in a single lane. One common method to index a library is adding barcodes during adapter ligation, so that the first five or six nucleotides of each read is the barcode. However, this strategy sacrifices read length, can increase the error rates at the 5^′^ or 3^′^ ends of reads [14], can perturb the calibration of the Illumina base calling algorithm (the HiSeq 2000 platform uses the first five nucleotides for calibration), and may lead to differential ligation efficiency and specificity among barcoded samples. Introducing barcodes during the final PCR amplification (Figure 1) bypasses these problems. The barcodes are then read using a separate primer and additional sequencing cycles after the insert has been sequenced (Figure 2). We modified the Illumina Multiplexing Sample Preparation Oligonucleotide Kit and used 12 barcoded primers to index 12 libraries at the final PCR step (Figure 1). Our protocol requires only 1 to 4 μg total RNA as starting material and takes no more than two days to complete.

**Table 1:** Comparison of library construction protocols

**Method**	**Time (days)**	**Total steps**	**Reagent cost ($)**	**Starting RNA (ng)**	**Use for small RNA**	**Kits available**	**Barcoding described**
This protocol	2	9	38	50	No	No	Yes
RNA ligation	8	19	250	1200	Yes	No	No
Illumina RNA ligation	5	16	240	100	Yes	Partially	No
Illumina RNA ligation-SPRI	4	12	220	100	Yes	Partially	No
SMART	5	8	80	100	Yes	No	No
Hybrid	5	13	90	500	Yes	No	No
NNSR	4	9	90	500	Unclear	No	No
NNSR, no Actinomycin D	4	9	90	500	Unclear	No	No
Bisulfite ‘S’	6	19	540	1000	No	Mostly	No
Bisulfite ‘H’	6	19	540	1000	No	Mostly	No
dUTP	5	17	430	200	No	Mostly	No
dUTP oligo(dT)	5	17	440	200	No	Mostly	No

Data for other methods from Levin [12]. Values only include steps after rRNA removal (this protocol) or poly(A) selection. dUTP: deoxyuridine triphosphate; NNSR: not not so random; SMART: switching mechanism at 5’ end of RNA transcript; SPRI, solid phase reversible immobilization.

## Methods

### Enzymes

TURBO DNase (2 U/μl, Ambion, Catalog Number AM2239; Life Technologies).

SuperScript III Reverse Transcriptase (200 U/μl, Catalog Number 18080–093; Life Technologies).

Ribonuclease H (RNase H) (2 U/μl, Invitrogen, Catalog Number 18021–014).

DNA Polymerase I (10 U/μl, New England Biolabs (NEB), Catalog Number M0209L; NEB, Ipswich, MA, USA).

T4 DNA Polymerase (3 U/μl, NEB, Catalog Number M0203).

DNA Polymerase I, Large (Klenow) Fragment (5 U/μl, NEB, Catalog Number M0210L).

T4 Polynucleotide Kinase (T4 PNK) (10 U/μl, NEB, Catalog Number M0236L).

Klenow Fragment (3^′^ to 5^′^ exo^–^) (5 U/μl, NEB, Catalog Number M0212L).

T4 DNA Ligase (600 U/μl, Enzymatics, Catalog Number L603-HC-L; Enzymatics, Beverly, MA, USA).

*Escherichia coli* UDG (5 U/μl, NEB, Catalog Number M0280S).

Phusion High-Fidelity DNA Polymerase (2 U/μl, NEB, Catalog Number M0530L).

AccuPrime *Pfx* DNA Polymerase (optional, 2.5 U/μl, Invitrogen, Catalog Number 12344–024).

### Buffers and Reagents

Ribo-Zero Magnetic Kit (Epicentre, Catalog Number MRZH116).

RNA Clean & Concentrator-5 (Zymo Research, Catalog Number R1015; Zymo Research, Irvine, CA, USA).

Random Primers (hexamers, 3 μg/μl, Invitrogen, Catalog Number 48190–011).

Deoxyadenosine triphosphate (dATP) (Bio Basic, Catalog Number DD0058; Bio Basic, Markham, Ontario, Canada).

Deoxycytidine triphosphate (dCTP) (Bio Basic, Catalog Number DD0058).

Deoxyguanosine triphosphate (dGTP) (Bio Basic, Catalog Number DD0058).

dTTP (Bio Basic, Catalog Number DD0058).

dUTP (Bio Basic, Catalog Number DM1244).

AMPure XP beads (Beckman Coulter, Catalog Number A63880; Beckman Coulter, Brea, CA, USA).

Elution Buffer for AMPure XP beads: 10 mM Tris–HCl (pH 8.5).

Zero Blunt TOPO PCR Cloning Kit (Invitrogen, Catalog Number K2800-20).

Luria-Bertani (LB) agar kanamycin plates: 1% (w/v) tryptone, 0.5% (w/v) yeast extract, 1% (w/v) NaCl, 1.5% (w/v) agar and 50 μg/ml kanamycin.

GoTaq Green Master Mix (Promega, Catalog Number M7122; Promega, Madison, WI, USA).

One Shot TOP10 Chemically Competent *E. Coli* (Invitrogen, Catalog Number C4040-10; or homemade [13]).

Super optimal broth with catabolite repression (SOC) medium: 0.5% (w/v) yeast extract, 2% (w/v) tryptone, 10 mM NaCl, 2.5 mM KCl, 10 mM MgCl_2_, 10 mM MgSO_4_ and 20 mM glucose (sterilize the glucose stock separately using a 0.2 μm filter, and then add it into the rest of ingredients, which should be sterilized by autoclaving).

70% (v/v) ethanol.

Second strand buffer/10× NEB Buffer 2: 500 mM NaCl, 100 mM Tris–HCl (pH 7.9), 100 mM MgCl_2_ and 10 mM DL-dithiothreitol (DTT).

10× T4 DNA Ligase buffer: 500 mM Tris–HCl (pH 7.5), 100 mM MgCl_2_, and 100 mM DTT, 10 mM ATP (note, ATP freshly added before use).

100 bp DNA ladder (for example, Fermentas, Catalog Number SM0241; Thermo Scientific, Waltham, MA, USA).

5× First strand buffer: 250 mM Tris–HCl (pH 8.3), 375 mM KCl, 15 mM MgCl_2_, 50 mM DTT.

2× Rapid ligation buffer: 132 mM Tris–HCl (pH 7.6), 20 mM MgCl_2_, 2 mM DTT, 15% PEG6000, 2 mM ATP (note, ATP freshly added before use).

Actinomycin D (optional, Sigma-Aldrich, Catalog Number A1410; Sigma-Aldrich, St Louis, MO, USA).

### Equipment

Water bath or heat block.

Magnetic stand for 1.5 ml centrifuge tubes.

Bench top centrifuge for 1.5 ml centrifuge tubes (17,000 × *g* required).

PCR thermal cycler.

NanoDrop (Thermo Scientific), or comparable low-volume spectrophotometer.

Bioanalyzer (optional; Agilent, Santa Clara, CA, USA).

37°C incubator.

Bench top vortexer.

### DNA oligonucleotides

#### Multiplexing adapters

Adapter oligo 1: 5^′^-pGAT CGG AAG AGC ACA CGT CT-3^′^

Adapter oligo 2: 5^′^-ACA CTC TTT CCC TAC ACG ACG CTC TTC CGA TCT-3^′^

#### PCR primers (barcode)

Primer 1: 5^′^-AAT GAT ACG GCG ACC ACC GAG ATC TAC ACT CTT TCC CTA CAC GAC GCT CTT CCG ATC T-3^′^

Primer 2 (primer with barcode, designed by combining Illumina Multiplexing PCR Primer 2.0 and PCR Index Primer into a single primer):

Primer 2–1: 5^′^-CAA GCA GAA GAC GGC ATA CGA GAT **CGT GAT** GTG ACT GGA GTT CAG ACG TGT GCT CTT CCG ATC T-3^′^

Primer 2–2: 5^′^-CAA GCA GAA GAC GGC ATA CGA GAT **ACA TCG** GTG ACT GGA GTT CAG ACG TGT GCT CTT CCG ATC T-3^′^

Primer 2–3: 5^′^-CAA GCA GAA GAC GGC ATA CGA GAT **GCC TAA** GTG ACT GGA GTT CAG ACG TGT GCT CTT CCG ATC T-3^′^

Primer 2–4: 5^′^-CAA GCA GAA GAC GGC ATA CGA GAT **TGG TCA** GTG ACT GGA GTT CAG ACG TGT GCT CTT CCG ATC T-3^′^

Primer 2–5: 5^′^-CAA GCA GAA GAC GGC ATA CGA GAT **CAC TGT** GTG ACT GGA GTT CAG ACG TGT GCT CTT CCG ATC T-3^′^

Primer 2–6: 5^′^-CAA GCA GAA GAC GGC ATA CGA GAT **ATT GGC** GTG ACT GGA GTT CAG ACG TGT GCT CTT CCG ATC T-3^′^

Primer 2–7: 5^′^-CAA GCA GAA GAC GGC ATA CGA GAT **GAT CTG** GTG ACT GGA GTT CAG ACG TGT GCT CTT CCG ATC T-3^′^

Primer 2–8: 5^′^-CAA GCA GAA GAC GGC ATA CGA GAT **TCA AGT** GTG ACT GGA GTT CAG ACG TGT GCT CTT CCG ATC T-3^′^

Primer 2–9: 5^′^-CAA GCA GAA GAC GGC ATA CGA GAT **CTG ATC** GTG ACT GGA GTT CAG ACG TGT GCT CTT CCG ATC T-3^′^

Primer 2–10: 5^′^-CAA GCA GAA GAC GGC ATA CGA GAT **AAG CTA** GTG ACT GGA GTT CAG ACG TGT GCT CTT CCG ATC T-3^′^

Primer 2–11: 5^′^-CAA GCA GAA GAC GGC ATA CGA GAT **GTA GCC** GTG ACT GGA GTT CAG ACG TGT GCT CTT CCG ATC T-3^′^

Primer 2–12: 5^′^-CAA GCA GAA GAC GGC ATA CGA GAT **TAC AAG** GTG ACT GGA GTT CAG ACG TGT GCT CTT CCG ATC T-3^′^

M13 Forward: 5^′^-GTA AAA CGA CGG CCA G-3^′^

M13 Reverse: 5^′^-CAG GAA ACA GCT ATG AC-3^′^

#### Procedure

##### rRNA depletion

High quality total RNA is essential for efficient rRNA removal. For example, in our hands, *Drosophila* RNA subjected to repeated freeze-thawing or treated with DNase cannot be efficiently depleted of ribosomal RNA.

1. Mix the Ribo-Zero magnetic beads by gently pipetting. For each total RNA sample, dispense 225 μl Ribo-Zero magnetic beads into an RNase-free 1.5 ml centrifuge tube.

2. Place the tube in the magnetic stand until the supernatant becomes clear, for approximately 1 minute.

3. With the tube still in the stand, discard the supernatant, which contains 0.1% sodium azide (chemical hazard: dispose of according to local regulations).

4. Add 225 μl RNase-free water to the tube, remove the tube from the magnetic stand, and mix the beads by gently pipetting.

5. Return the tube to the magnetic stand, wait until the solution becomes clear, and discard the water.

6. Resuspend the beads in 65 μl Ribo-Zero magnetic bead suspension solution and 1 μl RiboGuard RNase Inhibitor (Illumina). Mix well by gently pipetting. Store the tube at room temperature until step 9.

7. In a 1.5 ml centrifuge tube, prepare the following mix:

4 μg fresh total RNA

4 μl Ribo-Zero ‘reaction’ buffer

10 μl Ribo-Zero rRNA removal solution

Add water to make a 40 μl total volume.

Store the unused Ribo-Zero rRNA removal solution and ‘reaction’ buffer at −80°C.

8. Gently mix the solution by pipetting and incubate at 68°C for 10 minutes, then incubate the tube at room temperature for 5 minutes.

9. Gently mix the magnetic beads from step 6 by pipetting and add the RNA solution from step 8 to the mixed beads. Using the same pipette tip, immediately mix the beads with the RNA by pipetting 10 times. Next, vortex the tube for 10 seconds at medium speed. Finally, incubate the mixture at room temperature for 5 minutes.

10. Vortex the tube at medium speed for 5 seconds and then incubate it at 50°C for 5 minutes.

11. After the 5 minutes incubation, immediately place the tube in the magnetic stand for 2 minutes.

12. Carefully remove the supernatant, about 84 μl, to a new 1.5 ml centrifuge tube and place it in the magnetic stand for 1 minute to get rid of the trace amount of leftover beads from the last step.

13. Pipette the supernatant into a new 1.5 ml centrifuge tube and add 16 μl water.

##### Size selection and DNase treatment

RNA Clean & Concentrator-5 is used to enrich for RNAs >200 nt, which also removes 5S rRNA and tRNA.

14. Mix 100 μl RNA binding buffer with 100 μl 100% ethanol. Add this 200 μl mixture to the 100 μl RNA from step 13.

15. Transfer the buffer/ethanol/RNA mixture into a Zymo-Spin IC column (Zymo Research) in a collection tube. Centrifuge at 17,000 × *g* for 1 minute. Discard the flow-through.

16. Add 400 μl RNA Wash Buffer to the column, spin at 17,000 × *g* for 1 minute. Discard the flow-through.

17. To degrade contaminating DNA, mix the following reagents (to handle multiple samples at one time, we prefer to prepare the pre-mix for easy operation and less pipetting variance among samples):

3 μl TURBO DNase (2 U/μl)

3 μl 10× TURBO DNase Buffer

24 μl RNA Wash Buffer and add the 30 μl mixture to the column.

Incubate the column at 37°C for 30 minutes. Centrifuge the column at 17,000 × *g* for 1 minute, and discard the flow-through.

18. Add 400 μl RNA Prep Buffer to the column, centrifuge at 17,000 × *g* for 1 minute, and discard the flow-through.

19. Add 800 μl RNA Wash Buffer to the column, centrifuge at 17,000 × *g* for 1 minute, and discard the flow-through.

20. Add 400 μl RNA Wash Buffer to the column, centrifuge at 17,000 × *g* for 1 minute, and discard the flow-through.

21. Centrifuge the column at 17,000 × *g* for 2 minutes.

22. To elute the RNA, replace the collection tube with a new 1.5 ml centrifuge tube, and then add 10 μl water to the column. Incubate at room temperature for 1 minute and centrifuge at 17,000 × *g* for 1 minute to collect the RNA/flow-through.

23. Take 1 μl RNA to measure the concentration using a NanoDrop spectrophotometer or comparable low volume instrument. Typically, the procedure yields approximately 10 to 20 ng/μl (A260/A280 = 1.96 to 2.17).

#### Library preparation

1. Fragment the RNA. At high temperature, the metal ions within the 5× first strand buffer will hydrolyze the RNA into short fragments. In a 0.2 ml tube, mix 4 μl rRNA-depleted total RNA with 4 μl of 5× first strand buffer. Place the tube into a PCR thermal cycler pre-heated to 94°C. Incubate for precisely 4 minutes and 50 seconds. Then quickly chill the tube on ice for at least 1 minute.

2. To reverse transcribe the RNA into first strand cDNA, add to the PCR tube:

1 μl dNTP mixture (dATP, dCTP, dGTP, dTTP, 10 mM each)

0.5 μl 100 mM DTT

1.5 μl 100 mM DTT

1 μl Random Primer (hexamers, 3 μg/μl)

7 μl water.

Incubate at 65°C for 3 minutes, and then quickly chill the tube on ice for 1 minute. Next, add:

1 μl SuperScript III Reverse Transcriptase (200 U/μl)

4 μg Actinomycin D (optional, may enhance strand specificity but decrease uniformity of strand coverage [12]).

Incubate at 25°C for 5 minutes, then at 50°C for 1 hour. Heat at 70°C for 15 minutes to inactive the reverse transcriptase. Finally, use 36 μl AMPure XP beads to purify the cDNA, eluting with 22 μl elution buffer. (A detailed protocol for purifying with AMPure XP beads follows this protocol.)

3. To convert the first strand cDNA to double-stranded cDNA incorporating dUTP instead of dTTP, add the following to the cDNA from step 2:

3 μl second strand buffer/10× NEB Buffer 2

2 μl dUTP mixture (20 mM dUTP, 10 mM dATP, dCTP, dGTP)

1 μl RNase H (2 U/μl)

2 μl DNA Polymerase I (10 U/μl)

0.5 μl 100 mM DTT.

Incubate at 16°C for 2.5 hours.

After incubation, purify the double-stranded cDNA using 45 μl AMPure XP beads and elute the cDNA into a 1.5 ml centrifuge tube using 33 μl elution buffer. After this, continue or store the sample at -20°C.

4. Repair the ends of the double-stranded cDNA. DNA Polymerase I, which is used for second strand cDNA synthesis, uses as primers the RNA leftover from RNase H digestion. Consequently, the double-stranded cDNAs generated in step 3 have 3^′^ overhanging ends. Step 4 converts the sticky ends into blunt ends. Add the following mixture to the DNA from step 3:

5 μl 10× T4 DNA Ligase buffer

2 μl dNTP mixture (10 mM each)

5 μl T4 DNA Polymerase (3 U/μl)

1 μl Klenow DNA Polymerase (5 U/μl)

5 μl T4 PNK (10 U/μl).

Incubate at 20°C for 30 minutes.

5. To establish a library with the narrow size range (200 bp to 350 bp) required for successful high throughput sequencing, the cDNA is purified using AMPure XP beads, which exploit the finding that carboxyl coated magnetic beads bind distinct DNA size ranges depending on polyethylene glycol (PEG) and salt concentration [17-19]. After end repair, mix the reaction with 35 μl AMPure XP beads and incubate at room temperature for 5 minutes. Then place the tube in the magnetic stand for 3 minutes. Transfer the supernatant into a new tube and discard the beads. To the new tube with the supernatant, add an additional 40 μl of AMPure XP beads and then follow the standard AMPure XP bead purification protocol, eluting the DNA with 33 μl elution buffer.

6. Tail the PCR products with adenosine to facilitate adapter ligation. We use Klenow Fragment with D355A and E357A mutations (Klenow 3^′^ to 5^′^ exo^–^, 5 U/μl), a DNA polymerase lacking both 3^′^ to 5^′^ and 5^′^ to 3^′^ exonuclease activities, to add a single adenosine to the 3^′^ ends of the DNA. To the DNA from step 5, add:

5 μl second strand buffer/10× NEB Buffer 2

1 μl dATP (10 mM)

3 μl Klenow 3^′^ to 5^′^ exo^–^ (5 U/μl)

9 μl water.

Incubate at 37°C for 30 minutes. After incubation, purify the DNA using 60 μl AMPure XP beads; elute with 24 μl elution buffer.

7. Add the Y-shaped adapters (oligonucleotide sequences © 2007–2011 Illumina, Inc. All rights reserved.). To prepare the Y-shaped adapter, mix 25 μl adapter oligo 1 and oligo 2 (each at 50 μM stock concentration). Heat at 95°C for 2 minutes, then ramp down slowly to room temperature. We usually heat the oligo mixture in an aluminum heat block for 2 minutes. Then remove the block from the heater and let it cool down to room temperature, for approximately 30 minutes. Ligate the adapters to the purified double-stranded cDNA by adding:

25 μl 2× rapid ligation buffer

1 μl adapter (10 μM)

1.5 μl T4 DNA Ligase (600 U/μl).

Incubate at room temperature for 15 minutes. After incubation, use 50 μl AMPure XP beads to purify the DNA. Elute the DNA with 30 μl elution buffer.

8. Treat with 5 U/μl UDG. Add 2 μl UDG to the DNA from step 7, and incubate at 37°C for 30 minutes.

9. PCR amplify the cDNA. Add the following mixture to the DNA from step 8:

10 μl 5× HF Buffer (Phusion Polymerase, NEB)

1 μl 10 μM PCR Primer 2 (one of the twelve to provide the barcode)

1.5 μl dNTP (10 mM each)

0.5 μl Phusion High-Fidelity DNA Polymerase (2 U/μl)

5 μl water.

Incubate the tube at 98°C for 40 seconds, 65°C for 30 seconds and 72°C for 30 seconds. After the incubation, pause the PCR machine, and then add 1 μl 10 μM PCR Primer 1. Continue the PCR with 10 cycles of 98°C for 10 seconds, 65°C for 30 seconds, 72°C for 30 seconds, followed by incubation at 72°C for 3 minutes. Then purify the library with 50 μl AMPure XP beads. Finally, elute the DNA with 20 μl elution buffer.

Alternatively, the PCR can be performed using AccuPrime *Pfx* DNA Polymerase:

5 μl 10× AccuPrime *Pfx* Reaction Mix (Invitrogen)

1 μl 10 μM PCR Primer 2 (one of the twelve to provide the barcode)

1 μl AccuPrime *Pfx* DNA Polymerase (2.5 U/μl)

11 μl water.

Incubate the tube at 95°C for 40 seconds, 65°C for 30 seconds and 68°C for 30 seconds. After the incubation, pause the PCR machine, and then add 1 μl 10 μM PCR Primer 1. Continue the PCR with 10 cycles of 95°C for 15 seconds, 65°C for 30 seconds, 68°C for 30 seconds, followed by incubation at 68 °C for 3 minutes. Then purify the library with 50 μl AMPure XP beads. Finally, elute the DNA with 20 μl elution buffer.

##### Quality control (optional)

A good RNA-Seq library should have a narrow size range, retain strand information, and contain little RNA contamination from other species and few inserts from ribosomal RNA. Since high throughput sequencing remains expensive and time consuming, assaying the quality of libraries before submitting a sample for sequencing is worthwhile, particularly when first establishing this workflow. Here, we provide two ways to test library quality: Bioanalyzer analysis and small-scale colony sequencing. Using only 1 μl of the library, Bioanalyzer analysis provides fast, sensitive and accurate information on insert size distribution. Small-scale colony sequencing provides information on insert identity.

1. Bioanalyzer analysis. Run 1 μl of RNA-Seq library on the Agilent High Sensitivity DNA chip to check the size distribution of the library.

2. Small-scale colony sequencing. Mix:

0.5 μl RNA-Seq library

0.5 μl salt solution

0.5 μl pCRII-Blunt-TOPO (Invitrogen)

1.5 μl water.

Incubate at room temperature for 5 minutes. Pipette the reaction into 50 μl TOP10 *E. Coli* competent cells. To mix, gently tap the tube five times (do not mix by pipetting to avoid breaking the fragile cells), then incubate on ice for 20 minutes. Heat shock at 42°C for 40 seconds. Immediately chill on ice for 2 minutes, and then add 200 μl room temperature SOC medium. Recover the cells at 37°C for 1 hour with orbital shaking (200 rpm).

3. Spread 60 μl of the bacterial suspension on an LB plate containing 50 μg/ml kanamycin, and incubate at 37°C overnight.

4. Pick 10 to 20 colonies from each library. Gently touch a 10 μl pipette tip to a single colony, and then pipette with 12.5 μl GoTaq Green Master Mix, 1 μl of M13 Forward and Reverse primer mix (5 μM each) and 11.5 μl water. Run the following PCR program:

94°C 5 minutes

94°C 30 seconds

55°C 30 seconds

72°C 40 seconds (go to step 2 for an additional 30 cycles)

72°C 7 minutes

4°C hold.

5. Run 5 μl of the PCR product on a 1.5% agarose gel to check the size and quality of the PCR product. Use a 100 bp DNA ladder as size markers.

6. Sanger-sequence each PCR reaction that produces a product of a single length, using the M13 Reverse primer.

##### Additional protocol *using AMPure XP beads to purify DNA*

1. Warm the AMPure XP beads to room temperature, mix well, and pipette the required volume from the stock bottle to the sample tube.

2. Mix the beads and DNA sample by gently pipetting, and then incubate at room temperature for 5 minutes.

3. Place the tube in the magnet stand for 5 minutes until the supernatant appears clear.

4. Discard the supernatant.

5. Keep the tube in the stand and add 180 μl of 70% (v/v) ethanol into the tube without disturbing the beads.

6. Wait for 30 seconds and discard the ethanol supernatant.

7. Repeat steps 5 and 6.

8. To remove any ethanol remaining on the sides of the tube, centrifuge the tube at 1000 × *g* for 1 minute.

9. Place the tube in the magnetic stand for 30 seconds, and then remove any residual ethanol using a 10 μl pipette.

10. Add the specified volume of elution buffer to the beads and pipette to mix.

11. Wait 3 minutes, and then place the tube in the magnetic stand for 3 minutes.

12. Use a 10 μl pipette to carefully transfer the eluted DNA to a new tube (sacrifice 1 to 2 μl to avoid carrying over any beads).

## Anticipated results

### Quality control

**Figure 3:**
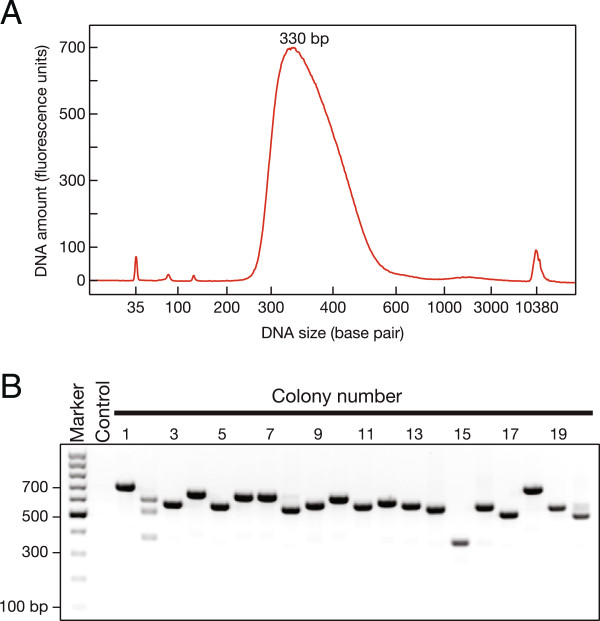
**Anticipated results.** (**A**) Bioanalyzer plot. (**B**) Agarose gel for small-scale colony sequencing. Control, a PCR reaction with no bacterial colony added.

Based on our experience, a good library will range in size from 300 to 500 bp, including 122 bp from the PCR primers plus 200 to 350 bp from the RNA inserts. Bioanalyzer analysis should show a peak at 320 to 330 bp (Figure 3A).

Small-scale colony sequencing can reveal the size and sequence of the inserts and barcodes for a small but representative sample of the library. When preparing libraries for the first time, sequencing 10 to 20 colonies per library serves to validate successful library construction. The PCR amplification products should be approximately 600 bp (244 bp from the pCRII-Blunt-TOPO vector, 122 bp from the PCR primers, plus the RNA insert; Figure 3B). Expect one or two colonies to lack inserts, giving a 366 bp PCR product (Figure 3B). Of the remaining 15 to 18 successfully sequenced colony PCR products, one or two may derive from rRNA.

### High throughput sequencing

The number of samples mixed in one sequencing lane depends on the genome size of the organism and the purpose of the research. To study low abundance RNAs from the repetitive region of the *Drosophila* genome, we usually pool four barcoded samples in a single lane of the HiSeq 2000 instrument, and sequence the libraries as 100 nt paired-end reads. We typically obtain >170,000,000 fragments per lane. For example, in one experiment, we obtained 175,991,972 fragments (for paired-end sequencing, each fragment has two reads, a total of 351,983,944 reads). Among them, 349,247,868 (99.2%) reads were successfully sorted by the barcodes.

Using TopHat [20-22] to map reads to the fly genome (parameters: tophat -i 50 -p 24 -library-type fr-firststrand -G gene.gtf -coverage-search -segment-length 25 -o output_directory_name), we typically achieve 90% mappability. For example, for a typical library, 91.7% of reads mapped to the fly genome. Among the mapped reads, only 4.03% were singletons (that is, only one of the paired reads in the fragment mapped); both reads mapped for the rest. Finally, more than 85% of mapped reads corresponded to protein-coding genes, and only 6.20% derived from non-coding RNAs such as rRNA, tRNA, snRNA or snoRNA. We have used this protocol to produce libraries of similar quality from wild-type and mutant mouse tissues, as well tissues from wild-type rat, chicken, and frog, demonstrating its general applicability.

## Abbreviations

dATP: Deoxyadenosine triphosphate; dCTP: Deoxycytidine triphosphate; dGTP: Deoxyguanosine triphosphate; DTT: DL-dithiothreitol; dTTP: Deoxythymidine triphosphate; dUTP: Deoxyuridine triphosphate; LB: Luria-Bertani; NEB: New England Biolabs; PCR: Polymerase chain reaction; PEG: Polyethylene glycol; piRNA: Piwi-interacting RNA; RNA-Seq: RNA sequencing; RNase H: Ribonuclease H; rRNA: Ribosomal RNA; snoRNA: Small nucleolar RNA; snRNA: Small nuclear RNA; SOC: Super optimal broth with catabolite repression; T4 PNK: T4 Polynucleotide Kinase; UDG: Uracil-DNA glycosylase.

## Competing interests

PDZ is a cofounder and member of the scientific advisory board of Alnylam Pharmaceuticals, Inc, and a member of the scientific advisory board of Regulus Therapeutics, LLC.

## Authors' contributions

ZZ and PDZ planned the experiments and wrote the manuscript. ZZ performed the experiments. All authors read and approved the final manuscript.
